# Molecular signatures of astrocytes and microglia maladaptive responses to acute stress are rescued by a single administration of ketamine in a rodent model of PTSD

**DOI:** 10.1038/s41398-024-02928-6

**Published:** 2024-05-25

**Authors:** Marta Valenza, Roberta Facchinetti, Carola Torazza, Claudia Ciarla, Maria Rosanna Bronzuoli, Matilde Balbi, Giambattista Bonanno, Maurizio Popoli, Luca Steardo, Marco Milanese, Laura Musazzi, Tiziana Bonifacino, Caterina Scuderi

**Affiliations:** 1 Department of Physiology and Pharmacology “Vittorio Erspamer”, SAPIENZA University of Rome, Rome, Italy; 2 Department of Pharmacy, Unit of Pharmacology and Toxicology, University of Genoa, Genoa, Italy; 3 Dipartimento di Scienze Farmaceutiche, Università Degli Studi di Milano, Milano, Italy; 4 IRCCS Ospedale Policlinico San Martino, Genoa, Italy; 5 School of Medicine and Surgery, University of Milano-Bicocca, Monza, Italy

**Keywords:** Molecular neuroscience, Pharmacology

## Abstract

Stress affects the brain and alters its neuroarchitecture and function; these changes can be severe and lead to psychiatric disorders. Recent evidence suggests that astrocytes and microglia play an essential role in the stress response by contributing to the maintenance of cerebral homeostasis. These cells respond rapidly to all stimuli that reach the brain, including stressors. Here, we used a recently validated rodent model of post-traumatic stress disorder in which rats can be categorized as resilient or vulnerable after acute inescapable footshock stress. We then investigated the functional, molecular, and morphological determinants of stress resilience and vulnerability in the prefrontal cortex, focusing on glial and neuronal cells. In addition, we examined the effects of a single subanesthetic dose of ketamine, a fast-acting antidepressant recently approved for the treatment of resistant depression and proposed for other stress-related psychiatric disorders. The present results suggest a prompt glial cell response and activation of the NF-κB pathway after acute stress, leading to an increase in specific cytokines such as IL-18 and TNF-α. This response persists in vulnerable individuals and is accompanied by a significant change in the levels of critical glial proteins such as S100B, CD11b, and CX43, brain trophic factors such as BDNF and FGF2, and proteins related to dendritic arborization and synaptic architecture such as MAP2 and PSD95. Administration of ketamine 24 h after the acute stress event rescued many of the changes observed in vulnerable rats, possibly contributing to support brain homeostasis. Overall, our results suggest that pivotal events, including reactive astrogliosis, changes in brain trophic factors, and neuronal damage are critical determinants of vulnerability to acute traumatic stress and confirm the therapeutic effect of acute ketamine against the development of stress-related psychiatric disorders.

## Introduction

Trauma- and stress-related disorders can result from one or more stressful events. Most people recover without specific intervention, but in some patients even a single traumatic experience can lead to psychiatric diseases, as in the case of post-traumatic stress disorder (PTSD) [1]. A large- scale study has recently shown that a diagnosis of a stress-related disorder is associated with a higher risk of death, including the one from unnatural causes, particularly suicide [2]. Improving mental health and suicide prevention is currently an international priority, in a way that the World Health Organization has set the ambitious goal of reducing global suicide mortality by one-third until 2030 [3]. Deepening our knowledge of the neurobiology of stress-related disorders is a critical step towards achieving this goal. In this context, a thorough understanding of the neurobiological underpinnings of resilience and vulnerability to stress is still lacking.

The short- and long-term consequences of acute traumatic stress can range from pro-adaptive to maladaptive depending on the characteristics of the traumatic event experienced (intensity, duration, lack of perceived control) and the person affected (genetic signature, previous life events, sex, education) [4]. Variables such as lack of social/family support, low socioeconomic status, and previous trauma exposure have been associated with exacerbation of trauma-related symptoms [5, 6]. When the intensity or duration of stress exceeds a certain individual’s threshold, activation of the stress response can be harmful, particularly to the brain, which is the master regulator of neuroendocrine and behavioral responses [4, 7].

Morphological and functional changes in glial cells have been associated with and may contribute to stress-related disorders [8–14], including PTSD [15–18]. Glial cells, particularly astrocytes and microglia, play various roles in the regulation of neuronal activity and synaptic plasticity. These cells are critical for maintaining brain homeostasis by modulating tissue architecture, ion fluxes, pH, neurotransmission, and neuroinflammation [19–22]. Although reactive astrogliosis, a common astrocytic change resulting from a pathological lesion, would be expected during a stress response, the literature shows rather opposite results. Reactive astrogliosis has been detected in depression and PTSD [23], with high expression levels of specific astrocytic proteins such as the cytoskeletal marker glial fibrillary acidic protein (GFAP), S100B, a Ca^2+^-binding protein that regulates neuronal firing at physiological levels but is neurotoxic when released in excessive amounts [24, 25], and connexin 43 (CX43), a component of the gap junctions responsible for the passage of gliotransmitters and other molecules that enables cell-cell communication [26]. Conversely, other groups have reported decreased morphometric properties of astrocytes and loss of astrocytic proteins, particularly GFAP, in regions involved in PTSD in both animals [16, 27] and humans [12, 28]. The involvement of astrocytes in modulating the stress response is also suggested by the available evidence on fibroblast growth factor 2 (FGF2). FGF2 is mainly synthesized in astrocytes, and its administration ameliorated anxiety and arousal symptoms in an animal model of PTSD [29]; conversely, other investigators have highlighted that increased FGF2 levels trigger reactive astrogliosis, suggesting a role for FGF-2 in CNS injury [30, 31]. Alterations in microglia morphology and function have also been demonstrated, particularly in models of chronic stress leading to depression-like behavior [32]. In response to chronic stress, microglia appear to be reactive and exhibit elevated levels of Iba-1, a protein involved in cytoskeletal movements, CD11b, a beta- integrin that is upregulated in reactive cells, and CD68, a lysosomal protein of microglia that is considered a marker for phagocytosis [33, 34]. Moreover, microglia in particular, but also astrocytes, are involved in immune responses and neuroinflammatory processes that are altered in numerous stress-related neurological and psychiatric disorders, including PTSD [15, 17, 18, 35–38]. Although the involvement of astrocytes and microglia has been documented in animal models of chronic stress that reproduce depression-like behaviors, their role in the acute stress response remains poorly studied.

Recent evidence suggests that subanesthetic doses of the fast-acting drug ketamine have beneficial effects in the treatment of stress-related disorders [39–42]. Ketamine is a non- competitive antagonist of the N-methyl-D-aspartate (NMDA) receptor that has been reported to rapidly alleviate symptoms in treatment-resistant MDD [43] and in PTSD patients [44–46] and has also shown resilience-promoting effects in animal models [47, 48]. We have recently shown that acute ketamine facilitates fear memory extinction and restoration of glutamatergic changes and dendritic atrophy in a rat model of PTSD [49].

In the present study, we used a recently validated rat model of resilience/vulnerability to acute inescapable footshock stress (FS) [50] to investigate the functional, molecular, and morphological determinants of the adaptive/maladaptive response to traumatic stress. Our experiments were performed in the rat prefrontal cortex (PFC) where we examined the density and function of astrocytes, microglia, and neurons. We investigated the PFC because we have previously shown that acute inescapable FS can induce both rapid and long-lasting structural and functional alterations in this brain region [49, 51–54]. Here, we also examined the effects of a single subanesthetic dose of ketamine administered shortly after stress exposure. We report differences in glial response, NF-κB activation, key brain factors related to synaptic architecture and neuronal density, and markers of neuroinflammation between vulnerable (FS-V) and resilient rats (FS-R). Ketamine restored many of the changes observed in FS-V, confirming its potential therapeutic value in psychiatric disorders triggered by a traumatic experience.

## Materials and methods

Detailed information is reported in the Supplementary Material.

### Animal procedures

Adult male Sprague-Dawley rats (175–200 g at the beginning of the protocol) were exposed to acute inescapable FS and deemed FS-R or FS-V as reported in Bonifacino et al. [50]. After having obtained the licenses required (N 521/2015-PR and 140/2014-B-DGSAF24898), all experimental procedures were performed according to the European Community Council Directive 2010/63/UE and the Italian D.L.26/2014.

### Drug and schedule of treatment

Racemic ketamine (MSD Animal Health, Milan, Italy) was dissolved in 0.9% saline and injected intraperitoneally at a subanesthetic dose of 10 mg/kg [49] 24 h after FS. Then, rats were left undisturbed in their cages until sacrifice, that had been scheduled 24 h after drug administration (i.e., 48 h after FS).

### Western blot

Sample preparation and western blotting (WB) were carried out according to our previously published protocols [55]. Details of the protocol, the antibodies used, and their dilutions are in the supplementary material and in Table S1. Target proteins were normalized to the total protein content.

### Real-time PCR

Real-time PCR was performed according to our published protocol [56]. Primer details and amplification conditions are listed in Table S2. Data were analyzed as ΔΔCT correcting for the actual efficiencies of the primers used [57].

### Immunofluorescence

Coronal slices (12 μm) containing the PFC were used for immunofluorescence, as previously reported [58]. Details of the antibodies and immunofluorescence conditions are listed in Table S3. Two independent investigators performed cell counting in 2–3 PFC fields acquired from each slice for a total of 4 slices/animal. Cell counts are reported as the number of positive cells per mm^2^.

### Statistical analysis

Statistical analysis was performed using GraphPad Prism software version 6.0 (GraphPad Software, San Diego, CA, USA). The normal distribution of the data was verified using Bartlett’s and Brown-Forsythe’s tests. Non-normally distributed data were analyzed using the non-parametric Kruskal-Wallis and Dunn’s post-*hoc* tests. Normally distributed data were analyzed by two-tailed unpaired Student’s *t*-test or one-way analysis of variance (ANOVA) or repeated measures ANOVA as appropriate. Upon detection of a significant main effect, multiple comparisons were carried out using Tukey’s post-hoc test.

Detailed results of the statistical analysis are reported in Supplementary Tables S4–S7. The exact sample size (*n*) for each experimental group is shown in each dot plot graph (each dot represents one animal).

## Results

### Acute footshock stress triggers a prompt astrocyte and microglia responses in the prefrontal cortex of vulnerable rats

We investigated whether resilience or vulnerability to acute stress is associated with changes in the density and functions of astrocytes and microglia in the PFC of rats, with particular attention to their contribution to neuroinflammation 24 h and 48 h after FS.

**Fig. 1:**
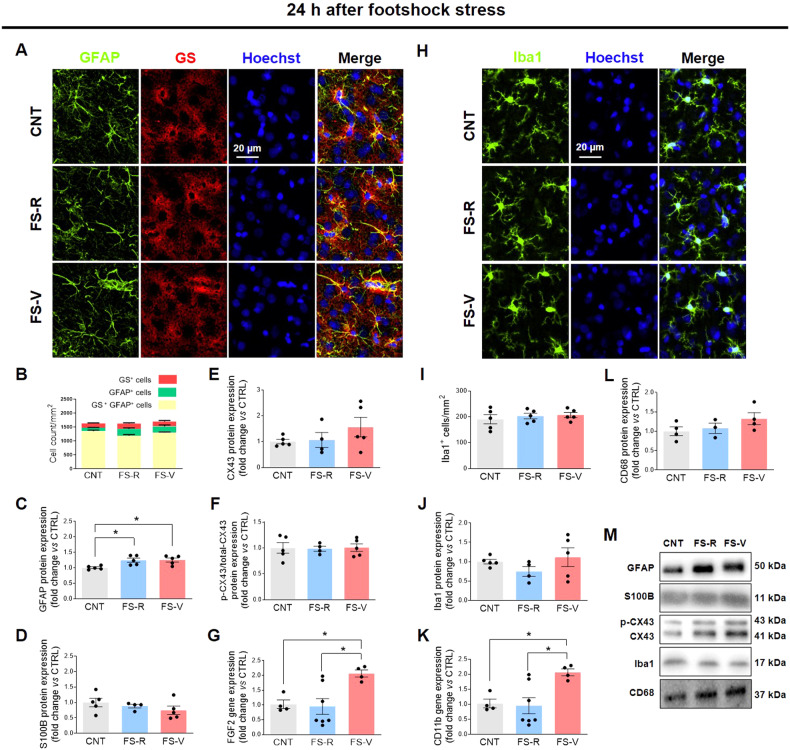

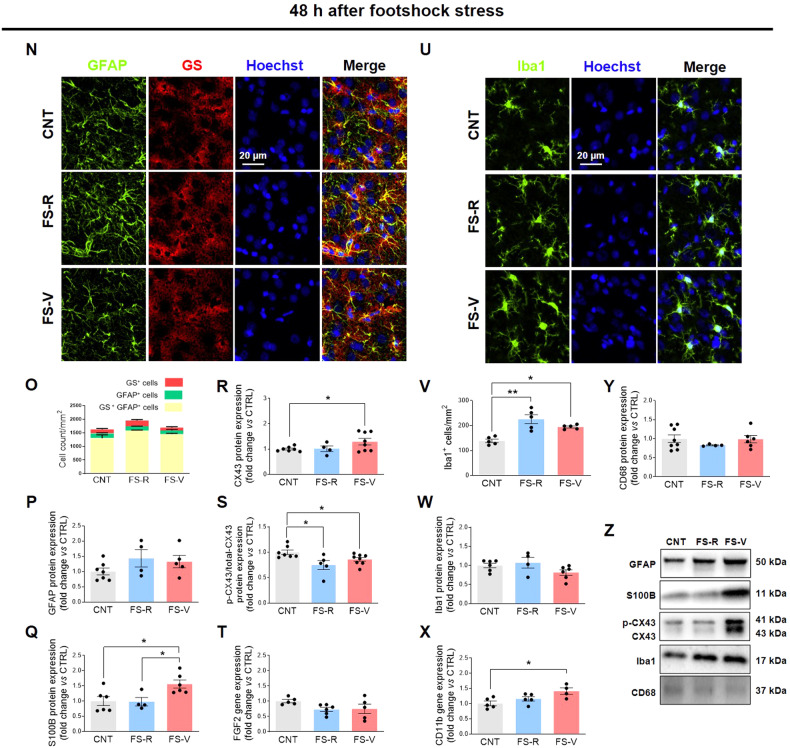
Astrocyte and microglia responses after acute footshock stress. Representative photomicrographs of PFC sections from CNT, FS-R, and FS-V sacrificed 24 h (**A**) and 48 h (**N**) after FS and stained for GFAP (*green*), GS (*red*), and Hoechst (*blue*). PFC cell density of GS^+^ cells, GFAP^+^ cells, and GS^+^GFAP^+^ cells measured 24 h (**B**) and 48 h (**O**) after FS; the graphs show mean ± sem of cell count/mm^2^ of *N* = 5 rats/group, *n* = 4 slices/rat, *n* = 2–3 images/slice acquired under a 20× objective. Cortical expression of GFAP (**C**, **P)**, S100B (**D**, **Q)**, CX-43 (**E**, **R)**, the ratio of phospho-CX43 to total CX-43 (**F**, **S**), and FGF2 (**G**, **T**) was analyzed in CNT, FS-R, and FS-V sacrificed either 24 h or 48 h after FS. Graphs show mean ± sem of *N* = 4–8/group. One-way ANOVA, Tukey’s post-hoc test: *p < 0.05. Representative photomicrographs of PFC sections from CNT, FS-R, and FS-V sacrificed 24 h (**H**) and 48 h (**U**) after FS and stained for Iba1 (*green*) and Hoechst (*blue*). PFC Iba1^+^cells density 24 h (**I**) and 48 h (**V**) after FS; the graphs show mean ± sem of cell counts/mm^2^ in *N* = 5 rats/group, *n* = 4 slices/rat, *n* = 2–3 images/slice acquired under a 20× objective. Cortical expression of Iba1 (**J**, **W**), CD11b (**K**, **X**), and CD68 (**L**, **Y**) was analyzed in CNT, FS-R, and FS-V sacrificed 24 h or 48 h after FS. Graphs show mean ± sem of *N* = 4–8/group. One-way ANOVA, Tukey’s post-hoc test: *p < 0.05, **p < 0.01. Representative images of western blotting bands of each target at 24 h (**M**) and 48 h (**Z**) after FS are included.

To assess astrocyte density, we examined cells expressing GFAP and glutamine synthetase (GS), the astrocytic enzyme responsible for the conversion of glutamate to glutamine. As shown in Fig. 1A, N, we identified three distinct astrocyte subpopulations: GS^+^ cells (red), GFAP^+^ cells (green), and GFAP^+^GS^+^ cells (yellow, as per co-localization). ANOVAs performed on cumulative cell density data showed no differences between experimental groups 24 h (Fig. 1B) and 48 h after stress (Fig. 1O). To study astrocyte reactivity, we analyzed the expression levels of GFAP, S100B, CX43, and FGF2 [59]. We observed a significant increase in GFAP protein expression at 24 h in both FS-V and FS-R compared to CNT, which persisted as a trend up to 48 h (Fig. 1C and 1P). No differences were observed in S100B protein expression at 24 h (Fig. 1D). In contrast, FS-V had higher S100B levels than CNT and FS-R at 48 h (Fig. 1Q). Moreover, we observed a trend toward increased expression of CX43 in FS-V 24 h after FS (Fig. 1E), which was significant 48 h after FS (Fig. 1R). CX43 opening is regulated by phosphorylation. Of note, when astrocytes are reactive, they can change CX43 expression and function, leading to dysregulation of permeability [60, 61]. CX43 phosphorylation was not significantly modified at 24 h (Fig. 1F), whereas a reduction of phosphorylation in FS-R and FS-V was observed 48 h after FS (Fig. 1S). Finally, FS-V showed higher FGF2 transcriptional levels than CNT and FS-R 24 h (Fig. 1G) but not 48 h (Fig. 1T) after FS.

To investigate the density and reactivity of microglia, we analyzed Iba-1, CD11b, and CD68 [27]. The number of Iba1^+^cells/mm^2^ (Fig. 1H, I) and Iba1 protein expression (Fig. 1J) did not differ between groups 24 h after FS. In contrast, 48 hours after FS, a stress-induced increase in microglial cell density was observed in both FS -R and FS -V compared to CNT (Fig. 1U, V), with no increase in Iba1 protein expression (Fig. 1W). Finally, CD11b gene expression was higher in FS- V both 24 h (Fig. 1K) and 48 h (Fig. 1X) after FS, whereas CD68 protein expression was not significantly changed at either time point (Fig. 1L, Y).

No changes in the morphology of astrocytes and microglia were observed at either time point (Fig. S1).

### Acute footshock stress increases specific proinflammatory mediators in the prefrontal cortex of vulnerable rats

**Fig. 2:**
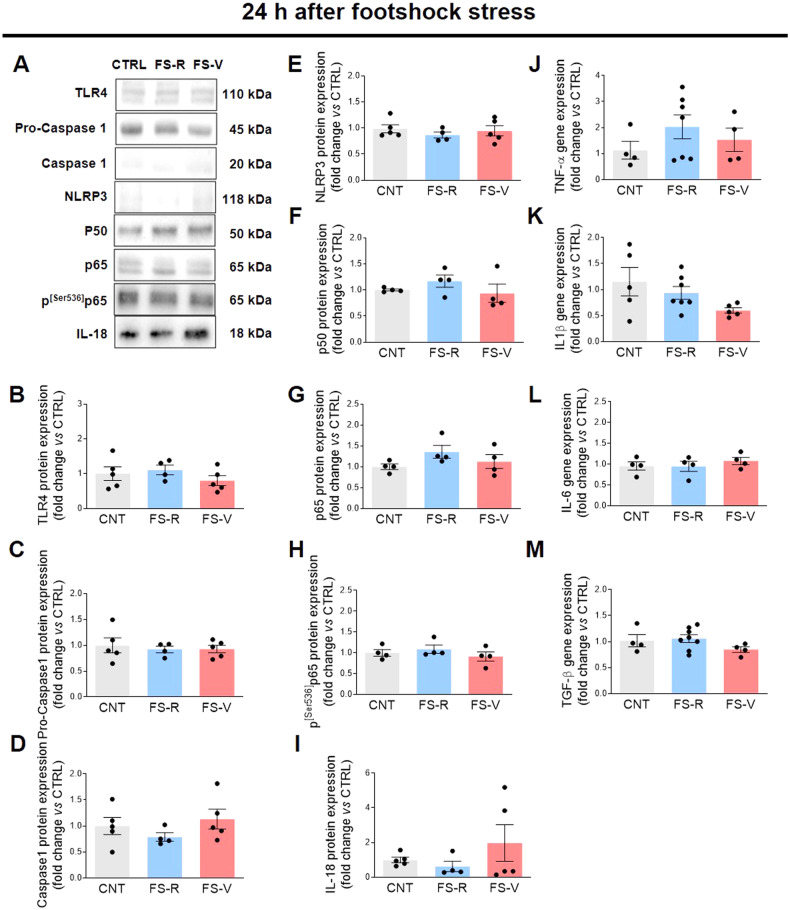

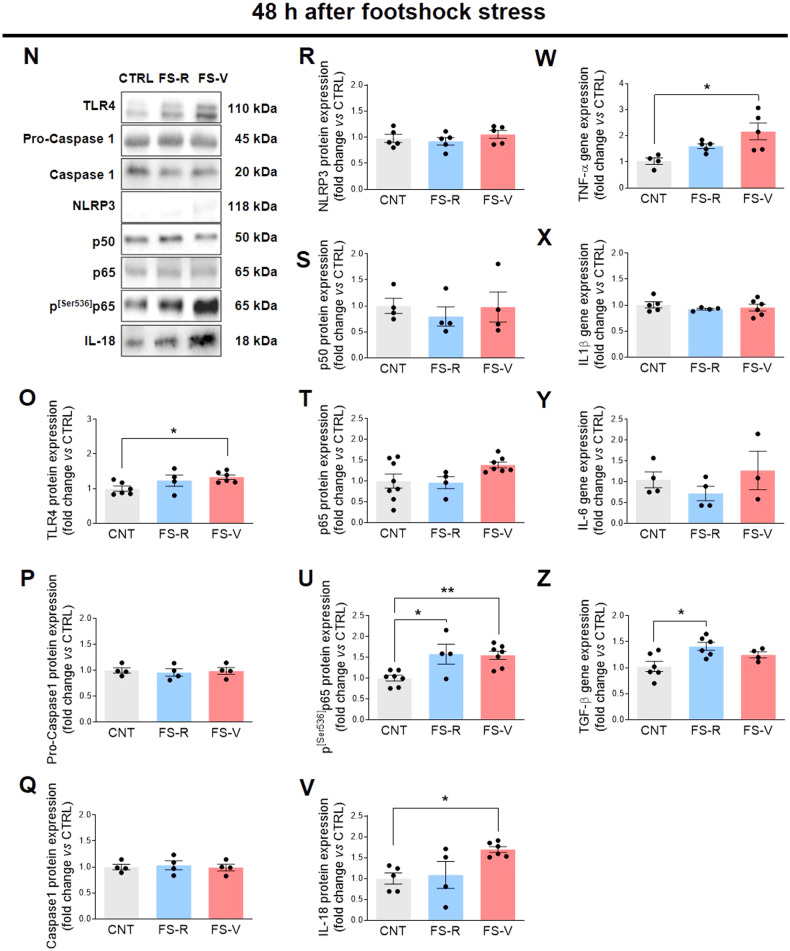
Effects of acute footshock stress on pro-inflammatory mediators. Representative images of western blotting bands of each target at 24 h (**A**) and 48 h (**N**) after FS. Cortical expression of TLR4 (**B**, **O**), pro-caspase 1 (**C**, **P**), caspase 1 (**D**, **Q**), NLRP3 (**E**, **R**), p50- NF-κB (**F**, **S**), p65-NF-κB (**G**, **T)**, p^[Ser536]^p65-NF-κB (**H**, **U**), IL-18 (**I**, **V**), TNF-α (**J**, **W**), IL-1β. (**K**, **X**), IL-6 (**L**, **Y**), and TGF-β (**M**, **Z**) was analyzed in CNT, FS-R, and FS-V sacrificed 24 h (**B**–**M**) or 48 h (**O**–**Z**) after FS. Graphs show mean ± sem of *N* = 3–8/group. One-way ANOVA, Tukey’s post-hoc test: *p < 0.05, **p < 0.01.

Consistent with astrocyte and microglia response, we also found a statistically significant increase in toll-like receptor (TLR)4 protein expression in FS-V compared to CNT 48 h after FS (Fig. 2O), but not at 24 h (Fig. 2B). TLRs are receptors of the innate immune system that are mainly located on microglia. In particular, TLR4 is essential for microglia to elicit responses to brain injury, as its activation induces downstream signaling pathways that promote the formation of proinflammatory mediators [62, 63]. In addition, we also found significant activation of NF-κB, a transcription factor composed of several DNA-binding proteins, including p65 and p50/p105. Through several phosphorylation steps, the complex can be activated and translocated to the nucleus, where it promotes transcription [64]. In particular, phosphorylation of p65 at serine 536 (p^[Ser536]^p65) is required for translocation to the nucleus and is a critical step for NF-κB to be considered active [65]. In our experimental conditions, we observed increased expression of p^[Ser536]^p65 in both FS-R and FS-V 48 h after stress (Fig. 2U), while protein expression of the constitutive isoforms p65 and p50 did not change (Fig. 2S, T). No changes were observed for p50, p65, and p^[Ser536]^p65 24 h after FS (Fig. 2F–H).

Since NF-κB activation induces transcription of proinflammatory molecules [66], we examined the expression of various cytokines both 24 h (Fig. 2I–M) and 48 h (Fig. 2V–Z) after FS. Consistent with NF-κB activation detected only 48 h after FS, we observed higher expression of the proinflammatory cytokines IL-18 (Fig. 2V) and TNF-α (Fig. 2W) in FS-V compared to CNT at this time point. Interestingly, we also observed a stress-induced increase in gene expression of the anti-inflammatory cytokine transforming growth factor (TGF)-β in FS-R (Fig. 2Z). Remarkably, there was no evidence either of activation of other proinflammatory signaling pathways, such as inflammasome NLRP3, procaspase 1, and caspase 1, or of an increase in the expression of canonical proinflammatory mediators, such as IL-1β and IL-6, 24 h (Fig. 2C–E and K, L) or 48 h (Fig. 2P–R and X, Y) after stress.

### Acute footshock stress leads to changes in dendritic and synaptic proteins and neurotrophins in the prefrontal cortex of vulnerable rats

**Fig. 3:**
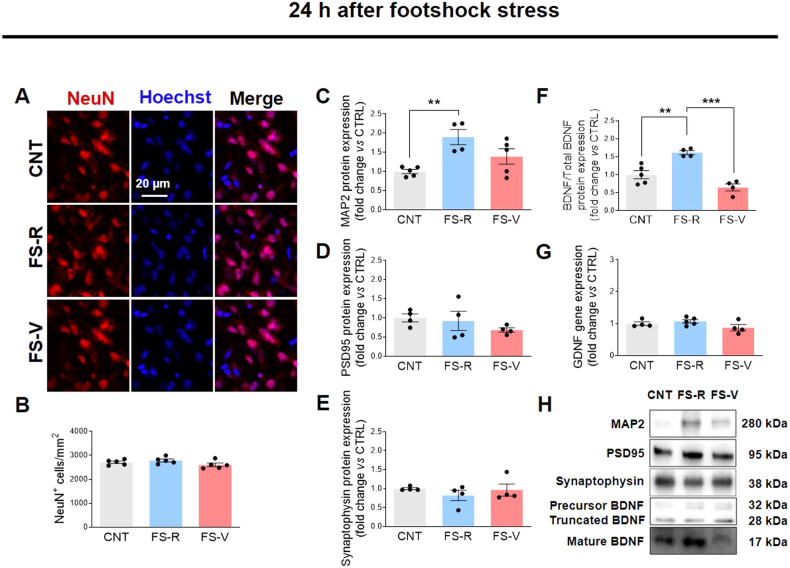

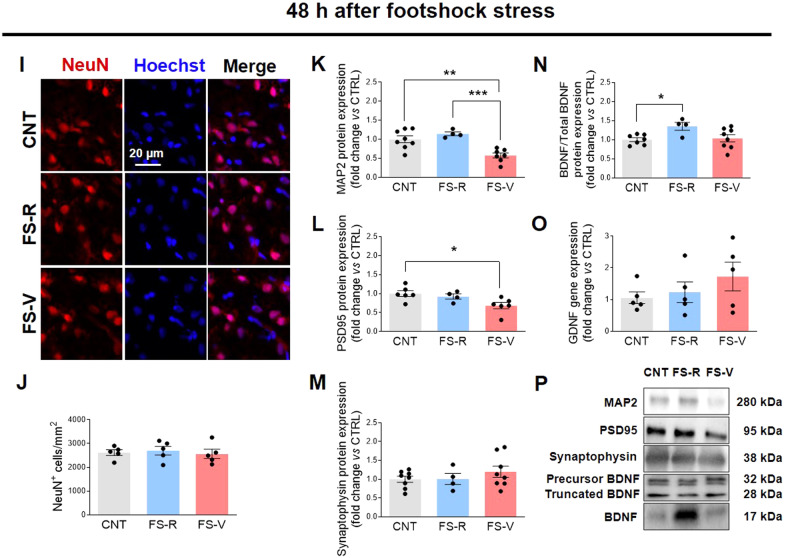
Effects of acute footshock stress on dendritic and synaptic proteins and neurotrophins. Representative photomicrographs of PFC sections from CNT, FS-R, and FS-V sacrificed 24 h (**A**) or 48 h (**I**) after FS and stained for NeuN (*red*) and Hoechst (*blue*). PFC NeuN^+^ cell density analyzed 24 h (**B**) and 48 h (**J**) after FS. Graphs show mean ± sem of cell count/mm^2^ in *N* = 5 rats/group, *n* = 4 slices/rat, *n* = 2–3 images/slice acquired under a 20× objective. Cortical expression of MAP2 (**C**, **K**), PSD95 (**D**, **L**), synaptophysin (**E**, **M**), the ratio of the mature isoform to total BDNF (**F**, **N**), and GDNF (**G**, **O**) was analyzed in CNT, FS-R, and FS-V sacrificed 24 h (**C**–**G**) or 48 h after stress (**K**–**O**). Graphs show mean ± sem of *N* = 4–8/group. One-way ANOVA, Tukey’s post-hoc test: *p < 0.05, **p < 0.01, ***p < 0.001. Representative images of western blotting bands of each target at 24 h (**H**) and 48 h (**P**) after FS are included.

Using the same model, we have previously shown that acute inescapable FS reduces the total length of apical dendrites of PFC neurons, in both FS-V and FS-R. In the same study, we found significant evidence for a selective reduction in the number of intersections in FS-V, suggesting neuronal retraction and simplification [50]. To verify the absence of neuronal death after FS, we examined cells expressing the neuronal marker NeuN at both 24 and 48 h and found no changes in neuronal density (Fig. 3A, B, I, J). We then analyzed the expression of microtubule-associated protein (MAP)2, the predominant cytoskeletal marker of neuronal dendrites [67], postsynaptic density protein (PSD)95, a scaffold protein of excitatory neurons that regulates the synaptic localization of various receptors and channels [68], and synaptophysin, an integral membrane glycoprotein found in presynaptic neurons and involved in vesicle formation and exocytosis [69]. As shown in Fig. 3C, FS-R exhibit higher MAP2 levels 24 h after FS than CNT. Even more interesting is the situation 48 h after FS, when we found significantly lower MAP2 levels in FS-V than in CNT and FS-R (Fig. 3K). No differences in PSD95 were observed 24 h after FS (Fig. 3D), whereas 48 h after stress FS-V expressed significantly lower levels of this protein than CNT (Fig. 3L). Finally, synaptophysin was not statistically altered at either time point (Fig. 3E, M).

Neurotrophins are crucial for dendrite remodeling induced by environmental factors, including stress [70]. Therefore, we measured the expression of the two major neurotrophins in the adult brain, brain-derived neurotrophic factor (BDNF) and glial cell-derived neurotrophic factor (GDNF), which are mainly expressed by neurons but also produced by glial cells in response to injury or brain damage [71, 72]. We found a marked and significant increase in BDNF protein expression in FS-R compared to CNT and a decrease in FS-V compared to FS-R 24 h after stress (Fig. 3F). The BDNF increase in FS-R persisted up to 48 h after stress (Fig. 3N). No significant changes were observed for GDNF gene expression at either time point (Fig. 3G, O).

### Acute ketamine rescues the molecular changes observed after footshock stress in the prefrontal cortex of vulnerable rats with no effects on resilient rats

**Fig. 4:**
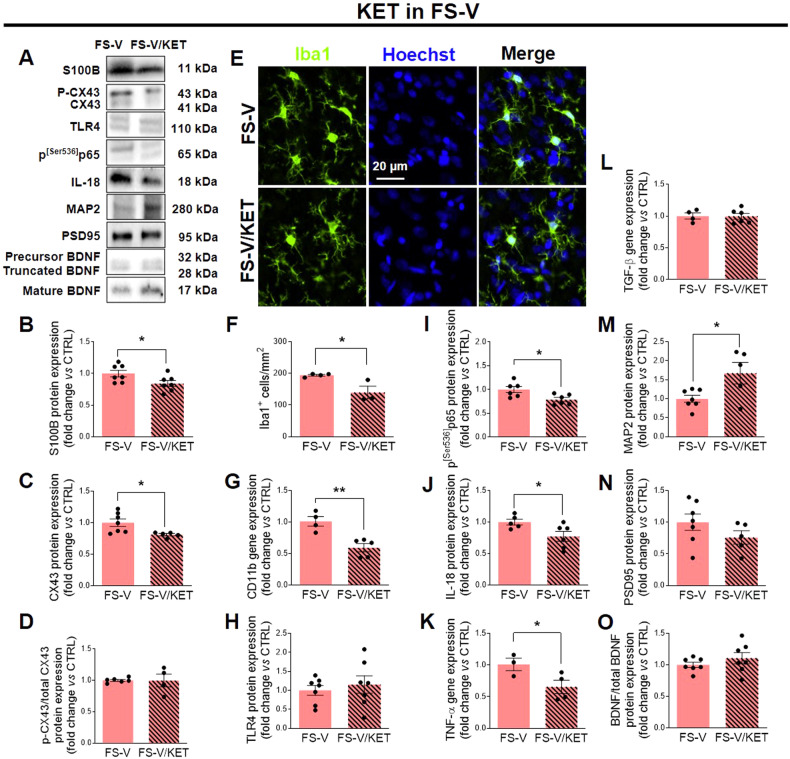

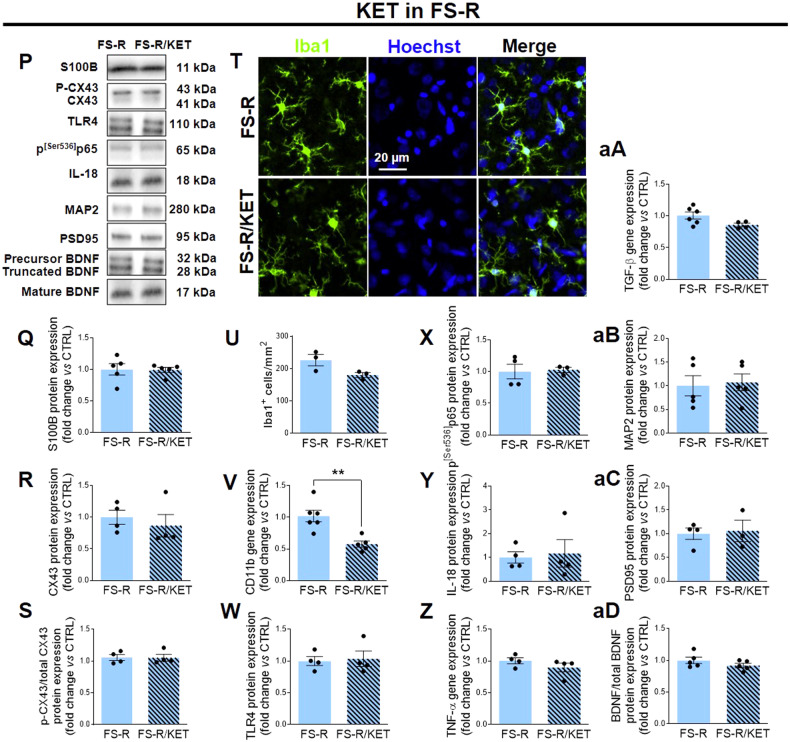
Effects of acute subanesthetic ketamine on glial and neuronal changes induced by acute footshock stress. Representative images of western blotting bands of each target studied in FS-V (**A**) and FS-R (**P**) treated with ketamine (Ket) or vehicle. Cortical expression of S100B (**B**, **Q**), CX43 (**C**, **R**), phospho-CX43/total CX-43 ratio (**D**, **S**). Graphs show mean ± sem of *N* = 4–7/group. Representative photomicrographs of PFC sections from FS-V (**E**) and FS-R (**T**), treated with Ket or vehicle, stained for Iba1 (*green*) and Hoechst (*blue*). PFC Iba1^+^ cell density measure (**F**, **U**); graphs show mean ± sem of cell count/mm^2^ in *n* = 5 rats/group, *n* = 4 slices/rat, *n* = 2–3 images/slice acquired under a 20× objective (**F**, **U**). Cortical expression of CD11b (**G**, **V**), TLR4 (**H**, **W**), phospho^[Ser536]^p65- NF-κB (**I**, **X**), IL-18 (**J**, **Y**), TNF-α (**K**, **Z**), TGF-β (**L**, **aA**), MAP2 (**M**, **aB**), PSD95 (**N**, **aC**), the ratio of mature BDNF to total BDNF (**O**, **aD**) was analyzed in FS-V (**G**–**O**) and FS-R (**V**–**aD**) following Ket or vehicle injection. Graphs show mean ± SEM of *N* = 3–7/group. Unpaired Student *t*-test: *p ≤ 0.05, ** *p* ≤ 0.01 Ket *vs* vehicle.

We have previously shown that an acute subanesthetic dose of ketamine administered soon after FS facilitates fear memory extinction and restores glutamatergic changes and dendritic atrophy in the PFC of rats exposed to FS [49]. Here, we found that FS-V treated with ketamine showed lower expression of astrocytic reactivity markers S100B (Fig. 4B) and CX43 (Fig. 4C) than vehicle- treated rats, without affecting CX43 phosphorylation (Fig. 4D). In addition, ketamine reduced the number of Iba1^+^ cells /mm^2^ (Fig. 4E,F) and CD11b expression (Fig. 4G), suggesting decreased microglia density and reactivity in FS-V treated rats. We did not detect any significant effects of ketamine administration on TLR4 expression levels (Fig. 4H). Conversely, ketamine decreased NF-κB activation in FS-V, as shown by low p^[Ser536]^p65 expression compared to vehicle (Fig. 4I). Treatment also decreased the levels of IL-18 (Fig. 4J) and TNF-α (Fig. 4K). In contrast, TGF-β was not affected (Fig. 4L). Moreover, ketamine significantly increased MAP2 (Fig. 4M) without affecting PSD95 and BDNF (Fig. 4N, O).

When the effects of ketamine were analyzed in FS-R, it was found that treatment did not alter the expression of the markers studied (Fig. 4Q–U, W–aD), with the exception of CD11b, whose expression was reduced by treatment (Fig. 4V).

## Discussion

Here, we report that acute, inescapable FS stress induces significant changes in the density and function of astrocytes and microglia as well as neuronal proteins. Moreover, we show that acute ketamine can rescue most of these changes. Our results are consistent with the literature supporting the therapeutic potential of ketamine in the treatment of stress-related psychiatric disorders.

We have previously shown that acute FS elicits rapid and sustained structural/functional synaptic changes in the rat PFC [50, 52, 53] and that administration of subanesthetic ketamine blocks sustained activation of excitatory synapses, abolishes dendrite shrinkage of pyramidal neurons, and facilitates the extinction of contextual fear memory [49]. Recently, we introduced the use of the sucrose intake test in rats exposed to acute FS as a rapid screening tool to distinguish acute stress-vulnerable from -resilient animals and to identify early determinants of a pro-adaptive or maladaptive stress response [50]. As previously shown, a single session of acute inescapable FS selectively triggers anhedonic-like behavior in a subpopulation of stressed rats, which are thus considered FS-V. We have also previously reported that the anhedonia observed in FS-V lasts for more than a week and is reversible, that glutamate release is differentially activated in neurons and astrocytes, and that FS-R and FS-V differ in the expression of astrocytic proteins involved in glutamate homeostasis, suggesting glial involvement in promoting a pro-adaptive or maladaptive response to FS [50]. A multi-omics study has shown that glial cells are the major contributors to the transcriptional bulk sequencing signal observed after acute stress [73].

In the present study, we further characterized the differences between FS-V and FS-R, suggesting a contribution of glial cells to the different behavioral phenotypes, and we demonstrated that a single administration of ketamine rescued most of the maladaptive changes observed in FS-V.

### A prompt cell response to FS stress in astroglia and microglia

We found that FS elicits a prompt response from glial cells, but this response differs significantly between FS-V and FS-R. We observed signs of astrocyte reactivity as early as 24 h after FS, as evidenced by the stress-induced increase in GFAP expression and the selective increase in FGF2 protein expression in FS-V. The latter is particularly interesting as FGF2 has been reported to mediate the glial response to brain injury by inducing more complex and hypertrophic astrocyte morphology in response to proinflammatory stimuli [30, 31, 56] and also acts as a reparative factor that suppresses astrocyte reactivity to ensure optimal neurological function [30]. In a recent in vitro study, exogenous FGF was shown to regulate astrocytic activation by reducing GFAP expression and lowering proinflammatory cytokines via activation of the upstream TLR4/NF-κB signaling pathway [74], which is closely associated with microglial reactivity (see below) [75, 76].

Forty-eight hours after FS, we detected a significant increase of S100B in FS-V compared to CNT and FS-R. This Ca^2+^-binding protein, which is mainly found in astrocytes, exerts paracrine trophic effects on various neuronal populations. S100B regulates several intracellular activities, including apoptosis, Ca^2+^ homeostasis, energy metabolism, inflammation and migration, proliferation, and differentiation. Astrocytes produce and secrete S100B, which has either neuroprotective or neurotoxic effects depending on its concentration. At higher concentrations, S100B contributes to reactive astrogliosis and positively regulates microglial activation [77]. Moreover, the observed changes in CX43 expression and phosphorylation underline the stress-induced reactivity of astrocytes. CX43 is the most abundant connexin in astrocytes, whose activity is negatively affected by inflammatory cytokines and has recently been linked to neurodegeneration, brain injury, and depression-like behavior [78, 79]. We observed a significant increase in CX43 in FS-V and a significant stress-induced decrease in CX43 phosphorylation. Fluctuations in the opening and permeability of this hemichannel are regulated by its phosphorylation, leading to the release of small mediators and ATP that can cause further glial activation and neuronal damage [80]. The decrease in CX43 phosphorylation may be due to the increase in FGF2 that we observed in FS-V 24 h after FS. Indeed, FGF2 can alter the phosphorylation status of CX43 and thus modify hemichannel opening [81].

Microglia showed early reactivity after acute stress with some differences between FS-R and FS-V. Already 24 h after FS and up to 48 h thereafter, CD11b levels were selectively increased in FS-V compared to CNT and FS-R. We also observed a stress-induced increase in the density of Iba1^+^ cells in both FS-R and FS-V 48 h after stress. We cannot determine the origin of the Iba1^+^ cells. As described in the literature, this finding could indicate the recruitment of microglia/macrophages from the periphery or the proliferation of resident microglia [13, 82]. Considering recent evidence showing that microglia recruit proinflammatory monocytes from the periphery in response to stress by releasing chemokines and cytokines such as CCL2, CXCL2, IL-1β, IL-6, and TNF-α [83], which we did not find altered (see below), we could speculate that proliferation rather than recruitment occurred in our model. However, regardless of the origin of these cells, our data suggest that microglia play a key role in the stress response. They express ion channels, neurohormone and neurotransmitter receptors that allow microglia to respond directly to changes in key mediators of the stress response, including glucocorticoids and catecholamines [13]. The arrival of stress-induced mediators in the brain triggers a microglial response that leads to activation of NFκB [84, 85], which occurs in different ways in FS-R and FS-V (see below).

### Different trajectories of stress response in vulnerable and resilient rats and the role of NF- κB

Both astrocytes and microglia contribute to the regulation of various brain functions, including neuroinflammatory processes [34]. Here, we also analyzed different effectors of neuroinflammation and found a selective proinflammatory pattern in FS-V 48 h after stress. Whereas no significant changes were detected 24 h after FS, we observed a stress-induced NF-κB activation 48 h later, which resulted in a proinflammatory response only in FS-V. Indeed, increased levels of TLR4 and proinflammatory cytokines such as IL-18 and TNF-α were detected only in FS-V. Conversely, in FS-R, we observed a marked increase in TGF-β, which has pleiotropic activities, including anti- inflammatory effects and the ability to reduce microglial reactivity.

It is worth noting that in addition to its role in regulating immune responses, NF-κB also controls many other non-immunological activities essential for cell survival and synaptic plasticity, including monitoring synaptic function and neuronal remodeling [86, 87]. NF-κB activation occurs in different models of chronic and acute stress as well as in patients exposed to stress or with major depression [88–90]. Also, NF-κB inhibitors attenuate some PTSD-like or depressive-like behaviors [89, 91]. Our data are consistent with these literature findings, as we observed stress-induced NF- κB activation in both FS-V and FS-R. However, the present results may suggest that this activation leads to a different outcome in the two subpopulations, namely a pro-reparative response in FS-R and an inflammatory response in FS-V. It is tempting to speculate that S100B in FS-V binds to TLR4 expressed in microglia, leading to NF-κB activation, which in turn increases transcription of stress-specific proinflammatory cytokines such as TNF-α and IL-18. The latter attracted our attention because there is growing evidence that IL-18 plays a specific role in mediating the brain response to stress [92, 93]. IL-18 is constitutively produced by cells of the immune system, but microglial cells, ependymal cells, and neurons also produce this cytokine. IL-18 can increase due to activation of the HPA axis and has been linked to brain disorders such as depression and cognitive impairment [93, 94]. In addition, IL-18-deficient mice show abnormalities in stress response [92]. These findings may open new avenues in the neurobiology of stress and suggest that IL-18 is a signal that mediates communication between the nervous, endocrine, and immune systems. Several molecular events occur in the FS-R brain, and we observed a pro-reparative response characterized by a glial response accompanied by an increase in protective mediators, such as TGF-β and BDNF.

### Selective changes in MAP2, BDNF, and PSD95 distinguish resilient from vulnerable rats

We have previously shown that stress alters neuronal/synaptic complexity in stressed animals. Indeed, we demonstrated that FS causes shortening and simplification of apical dendrites of pyramidal neurons of layers II–III of the prelimbic PFC which was measurable as early as 24 h and persisted up to 14 days after stress [49, 50]. Our previous data showing a greater reduction in the number of intersections selectively in FS-V suggest subtle stress-induced differences in dendritic remodeling [50]. We also showed that the total length of apical dendrites and the number of dendritic branches in PFC layers II–III decreased in both FS-R and FS-V rats. Sholl analysis revealed a significant decrease in the number of intersections between 120 and 180 μm from the soma only in FS-V animals compared to CNT [50]. In agreement with our previous results, here, we found a significant increase in the dendritic marker MAP2 and the neurotrophin BDNF exclusively in FS-R (24 h after stress that persisted up to 48 h after stress), suggesting an activation of trophic mechanisms that presumably counteract the effects of stress. In contrast, a decrease in MAP2 and PSD95 expression was measured in FS-V 48 h after stress, suggesting dendrite simplification and synaptic loss.

Overall, our data suggest that microglia could play a role in the morphological effects of stress. Indeed, microglia physiologically exhibit phagocytic activity that may be responsible for neuronal remodeling in the adult brain [95]. Microglia reactivity, usually associated with the release of proinflammatory cytokines, has been linked to pathological dendritic remodeling [17, 96, 97]. Therefore, it is reasonable to speculate that microglia are involved in the morphological and functional neuronal changes observed here in FS-V. Further studies are needed to verify this hypothesis. Consistent with our results, in another mouse model of FS-induced PTSD, microglia were responsible for the reduction in cortical dendritic branches and spine density [98].

BDNF is one of the most studied molecules in biological psychiatry, and its involvement in the stress response and associated mental disorders is widely recognized [99–101]. There is growing evidence that stress targets BDNF, suggesting that the BDNF/TrkB pathway is critical for stress- related depression and anhedonia [102]. BDNF is a common downstream mediator of environmental factors that enhance anxiety- and depressive-like behaviors [103, 104]. The BDNF increase in FS-R and the decrease in FS-V suggests a crucial role of this neurotrophin in regulating the stress response. Notably, we previously found a similar selective increase of BDNF in FS-R from a chronic stress protocol [105]. We hypothesize that FS-R activate complex cellular and molecular responses to cope with stress, which is not the case in FS-V.

### Acute subanesthetic ketamine rescues glial and neuronal changes in vulnerable rats

We have previously shown that ketamine abolishes the increase in depolarization-evoked glutamate release and peak amplitude of spontaneous excitatory postsynaptic currents in the PFC of stressed animals and rescues stress-induced dendritic retraction of pyramidal neurons [49].

Here, we show that acute ketamine is able to decrease parameters of reactive astrogliosis such as S100B and CX43, microglia recruitment/proliferation and reactivity such as Iba1 and CD11b, activation of NF-κB signaling, and expression of proinflammatory cytokines IL-18 and TNF-α, all changes that were selectively altered in FS-V by FS. We also observed that ketamine led to increased expression of the dendritic protein MAP2, suggesting its neuroprotective effects. Collectively, our data suggest that a single ketamine administration after FS exposure prevents the harmful glial changes in FS-V thus protecting neurons and promoting a resilient-like molecular environment.

Future studies should elucidate how ketamine exerts these effects among the many mechanisms of action described [106]. Whatever the case, the anti-inflammatory effects of ketamine are not new [107, 108]; Zanos and colleagues reported that ketamine exerts immunomodulatory but not immunosuppressive effects [39], which is consistent with our findings.

## Conclusions

Even a single exposure to traumatic stress can trigger psychiatric symptoms in vulnerable individuals and lead to psychiatric disorders such as major depression or PTSD. Therefore, there is an urgent need to identify the early signs of maladaptive responses to stress and to find effective and fast-acting therapies. This study is a step towards understanding the neurobiology of stress, deciphering the different trajectories that vulnerable and resilient individuals follow to cope with stress, and providing clues to a potential therapeutic strategy.

The new data presented here and summarized in Table 1 provide the basis for future studies to decipher glial mechanisms in adaptive and maladaptive responses to stress.

**Table 1:** Results of the targets analyzed in the PFC of CNT, FS-R, and FS-V 24 and 48 h after FS and the effects of ketamine on each target.

Target (alphabetical order)	FS-R 24 h after FS	FS-R 48 h after FS	KETAMINE effect in FS-R	FS-V 24 h after FS	FS-V 48 h after FS	KETAMINE effect in FS-V
BDNF/total BDNF	↑	↑	NO	–	–	NO
CD11b	–	–	YES	↑	↑	YES
CX43	–	–	NO	–	↑	YES
FGF2	–	–	NO	↑	–	NO
GFAP	↑	–	NO	↑	–	NO
Iba1^+^cells	–	↑	NO	–	↑	YES
IL-18	–	–	NO	–	↑	YES
MAP2	↑	–	NO	–	↓	YES
p-CX43/CX43	–	↓	NO	–	↓	NO
_p_[Ser536]_-p65_	–	↑	NO	–	↑	YES
PSD95	–	–	NO	–	↓	NO
S100B	–	–	NO	–	↑	YES
TGF-β	–	↑	NO	–	–	NO
TLR4	–	–	NO	–	↑	NO
TNFα	–	–	NO	–	↑	YES

**Fig. 5:**
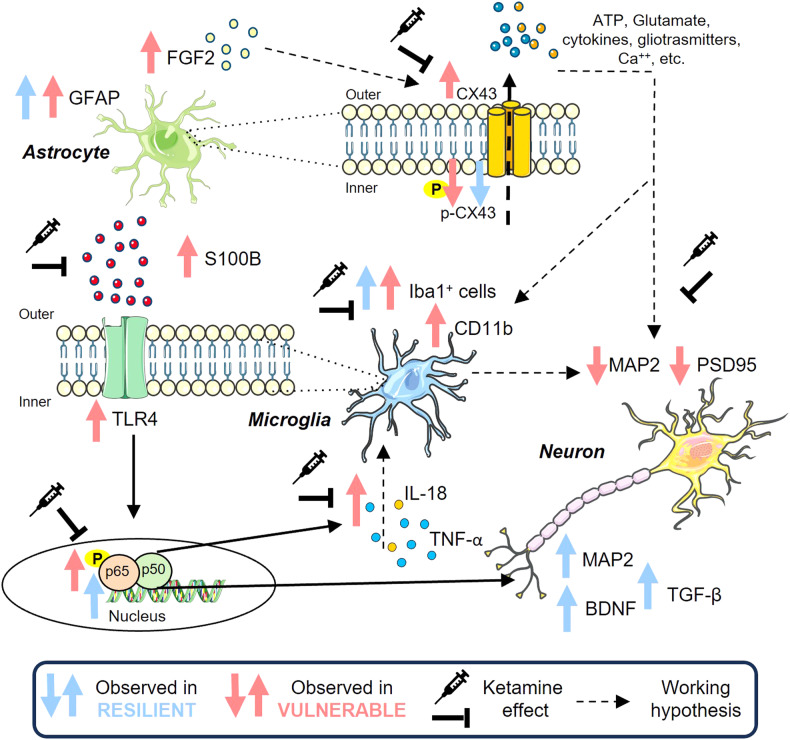
Schematic diagram of the proposed neurobiological differences between rats deemed resilient (*blue*) or vulnerable (*pink*) to acute FS and the effect of ketamine (*black*). We hypothesize that astrocytes react to FS by producing high levels of S100B, which can interact with the microglial TLR4, one of its physiological targets, which we found increased in FS-V. This interaction would activate NF-κB, increasing specific proinflammatory cytokines such as TNF-α and IL-18. These cytokines, together with FGF2, mainly produced by reactive astrocytes, may cause a decrease in phosphorylation of CX43, which is responsible for the altered opening of astrocytic hemichannels, leading to the release of gliotransmitters and proinflammatory molecules. Such a process would result in a toxic environment harmful to neurons, as evidenced by the decrease in MAP2 and PSD95 in FS-V. Instead, FS-R, the molecular responses are different, and the most apparent difference is the increase in TGF-β, BDNF, and MAP2. Furthermore, ketamine rapidly attenuates glial changes and NF-κB activation, IL-18 and TNF-α expression, and promotes neuronal health in FS-V.

Based on the results, we speculate that the selective glial reactivity observed in FS-V rats may explain the dendritic atrophy seen in the PFC as synaptic remodeling activity driven by microglia is stimulated by inflammatory processes (see Fig. 5). In contrast, glial response in FS-R rats appears to be balanced by neurotrophic mechanisms, restoring homeostasis. This could have both therapeutic and diagnostic implications. Glial or neuroinflammatory parameters could become biomarkers that can be used to discriminate between resilient and vulnerable individuals in a population exposed to a traumatic event. Moreover, our data from this and previous studies suggest that the administration of ketamine to vulnerable individuals may help to interrupt the spiral of molecular changes triggered by traumatic stress [39, 106]. Ketamine has been suggested as an effective treatment for PTSD and it should be further investigated [42, 109, 110], as clinical trials have reported a reduction in symptom severity in PTSD patients treated with this drug at subanesthetic doses [45, 111–113].

We are aware of the limitations of this study, including the issue of sex. It is well known that females are more susceptible to stress-related adverse outcomes. However, we decided to use only male rats because hormonal fluctuations influence many brain functions, adding supplementary variables. The use of female animals will undoubtedly be our next step, taking advantage of the knowledge we have already gained. Moreover, we focused our studies on PFC and neglected other brain regions affected by stress that should be investigated in the future.

Overall, the present work suggests a link between vulnerability/resilience to acute stress, changes in astrocyte and microglial functions, BDNF availability, and neuronal changes. In contrast, acute ketamine restores brain homeostasis by controlling the balance between neuroinflammation and dendritic arborization.

### Supplementary information

Supplementary Information

## Data Availability

Data is available on request from the corresponding author.
