# Supplementary material for: Molecular signatures of astrocytes and microglia maladaptive responses to acute stress are rescued by a single administration of ketamine in a rodent model of PTSD
Source: Transl Psychiatry. 2024 May 25;14:209. doi: 10.1038/s41398-024-02928-6 (PMC11127980; doi:10.1038/s41398-024-02928-6)
Supplement: Supplementary file 1 — Supplementary Information [file 41398_2024_2928_MOESM1_ESM.docx]

**Supplementary Information**

This file includes:

Supplementary Methods including Tables S1, S2, S3.

Supplementary Results (Tables S4, S5, S6, and S7)

Supplementary figure 1

**Supplementary Methods**

**Animal procedures**

*Animals*

Adult male Sprague-Dawley rats (175–200 g at the beginning of the protocol) were group-housed (except during the sessions of sucrose intake) with free access to food and water on a 12/12 h light/dark schedule (lights on at 7:00 AM), in a temperature- and humidity-controlled facility. After obtaining the required licenses (N 521/2015-PR and 140/2014-B—DGSAF24898, all experimental procedures were performed following the European Community Council Directive 2010/63/UE and the Italian D.L.26/2014.

*Footshock stress protocol*

Animals were subjected to a single session of acute inescapable footshock stress (FS) consisting of intermittent shocks (0.8 mA) for 40 min (20 min total of actual shock with random intershock length between 2 and 8 s), as previously reported [1]. The FS box was connected to a scrambler controller (LE 100-26, Panlab) that delivered intermittent shocks to the metal floor. The control animals (CNT) were left undisturbed in their home cages.

*Sucrose intake test and identification of resilient and vulnerable rats*

Rat sucrose intake was evaluated as in Bonifacino, Mingardi, Facchinetti, Sala, Frumento, Ndoj et al. [1]. After a 1 one week of acclimatization period, rats were habituated to a palatable sweet solution by removing the water bottle and exposing them to two bottles containing a 1% sucrose solution for 2 h. From the following day, the rats were housed individually and provided with two bottles, one with 1% sucrose and one with tap water, without food pellets, for 1 h. The position of the bottles was reversed after 30 min. This procedure was repeated twice a week for 4 weeks. The animals were never deprived of food and water before the test.

The average amount of sucrose solution drunk by each animal was calculated and defined as the baseline sucrose intake. After 4 weeks, the animals were randomly assigned to receiving a FS or left undisturbed in their home cages (CNT). The sucrose intake test began 23 h after the start of FS and the percentage sucrose intake compared to baseline was calculated for each animal. Animals in which sucrose intake decreased by at least 25% from baseline were considered anhedonic and classified as vulnerable (FS-V), while all others were defined as resilient (FS-R). The sucrose intake test was then repeated 48 h after FS. Rats were sacrificed by beheading 24 h or 48 h after FS. Rats were randomly assigned to receiving ketamine (10 mg/kg) or vehicle 24 h after FS and were sacrificed 24 h later (i.e. 48 h after FS). Investigators were not blinded to the group allocation.

**Western Blot**

PFC was homogenized in ice-cold hypotonic lysis buffer containing 50 mM Tris/HCl pH 7.5, 150 mM NaCl, 1 mM ethylenediaminetetraacetic acid (EDTA), 1% Triton X-100, 1 mM phenylmethylsulfonyl fluoride (PMSF), 10 μg/ml aprotinin, and 0.1 mM leupeptin (all from Sigma-Aldrich, Saint Louis, MO, USA) were added and allowed to stand for 40 min at + 4 °C. After centrifugation for 30 min at 14000 rpm (19721 x *g*), the supernatant was collected and stored at − 80 °C. Protein concentration was calculated by BCA assay (Thermo Fisher, Waltham, MA USA). Thirty micrograms of proteins were resolved on precasted 4-20% acrylamide gradient SDS-PAGE gels and then transferred to nitrocellulose membranes using TurboBlot (Bio-Rad, Hercules, CA, USA). Nonspecific binding of antibodies was avoided by using an appropriate blocking solution for 1 h at room temperature. Then, incubation with primary antibodies was performed overnight at + 4 °C. The next day, membranes were rinsed with Tris Buffered Saline (TBS)-Tween 0.05% and incubated with specific secondary horseradish peroxidase (HRP)-conjugated antibodies for 1 h at room temperature. After additional rinses with TBS, signals were detected using an enhanced chemiluminescence (ECL) kit (GE Healthcare Life Sciences, Milan, Italy), visualized using a Chemidoc XRS +, and quantified with Image Lab software (Bio-Rad). Values were normalized to those of total protein. All the experimental conditions used are listed in Table S1.

**Real-time PCR**

Total mRNA from PFC was isolated using TRI-Reagent (Sigma-Aldrich, Saint Louis, MO, USA) and quantified using the D30 BioPhotometer spectrophotometer (Eppendorf AG, Hamburg, Germany). One microgram of mRNA was reverse transcribed using a first-strand cDNA synthesis kit in the presence of 0.2 μM oligo(dT) and 0.05 μg/μL random primers (Promega, Promega Corporation, WI, USA). The thermal protocol included one step at 25 °C for 10 min and one at 72 °C for 65 min. Primers and cDNA were mixed with the iTaq Universal SYBR Green Supermix (Bio-Rad, Hercules, CA, USA). The thermal protocol used for amplification included an initial step at 95 °C for 3 min and 40 cycles with one step at 95 °C for 10 s and one at 60 °C for 30 s, using the CFX96 Touch thermal cycler (Bio-Rad). All primer sequences and details are listed in Table S2. Melting curve analysis of the amplification products was performed at the end of the reaction, increasing the temperature from 65 to 95 °C in 0.5 °C increasing steps. The amount of each target amplicon was normalized to the mean value of mRNA from three reference genes: TATA-box binding protein (TBP), glyceraldehyde-3-phosphate dehydrogenase (GAPDH), and hypoxanthine-guanine phosphoribosyl transferase (HPRT). All samples were run concurrently in triplicate. Data were analyzed as ΔΔCT corrected for the actual efficiency of the primers used [2].

**Immunofluorescence**

Upon rat sacrifice, the brains were immediately extracted, flash frozen using 2-methylbutane, and stored at −80 °C. Coronal slices (12 μm thickness) containing the PFC were obtained using a cryostat (Thermo Fisher Scientific, Waltham, MA, USA) and mounted on microscope slides. The sections were fixed in 4% paraformaldehyde in 0.1 M phosphate buffer saline (PBS) for 10 min at 4 °C. Sections were permeabilized and non-specific binding was blocked with the appropriate solution for 1 h at room temperature. Then, slices were incubated overnight with the proper primary antibody at + 4°C. Then, the sections were rinsed with PBS 1X and incubated with the corresponding secondary antibody for 2 h at room temperature. Cell nuclei were stained with Hoechst (1:5000, Thermo Fisher Scientific). After rinsing with PBS 1X, the slices were mounted with Fluoromount aqueous mounting medium (Sigma-Aldrich). Experiments were carried out by two blinded investigators. All experimental conditions used are listed in Table S3.

**Morphological analysis of astrocytes and microglia**

Using Fiji software, we performed morphometric characterization of GFAP^+^ and Iba1^+^ cells as reported by Torres-Platas et al [3]. The length of branches was measured by tracing a freehand line on the cell primary processes. The degree of branching was assessed by counting the total number of terminals of each cell. The soma diameter of Iba1^+^ cells was measured by drawing a straight line crossing the cells at their shortest axis. Experiments were carried out by a blinded investigator.

**Statistical Analysis**

Statistical analysis was performed using GraphPad Prism software version 6.0 (GraphPad Software, San Diego, CA, USA). The Jackknife method was used to detect outliers (cutoff: ±2.5). The normal distribution of the data was verified using Bartlett's and Brown- Forsythe’s tests. Normally distributed data were analyzed by two-tailed unpaired Student’s t-test or one-way analysis of variance (ANOVA) or repeated measures ANOVA as appropriate. Upon detection of a significant main effect, multiple comparisons were carried out using Tukey's post-hoc test. Non-normally distributed data were analyzed using the non-parametric Kruskal- Wallis and Dunn’s post-hoc tests. F test was applied to compare variances within each group, p>0.05. The number of animals used in each experiment is indicated in the figure legends and sample size was calculated based on previous experiments in order to have a power >80% to detect differences >30% at SD of 25% and alpha error of 5%.

**Table S1. List of antibodies and Western blotting conditions**

| **Primary antibody** | **Brand and Catalog #** | **Dilution** | **Secondary antibody** | **Brand and Catalog #** | **Dilution** |
| --- | --- | --- | --- | --- | --- |
| α-BDNF | Bioss,  MA, USA  bs-4989R | 1:1000, 5% milk in TBS-T 0.1% | HRP conjugated goat anti-rabbit IgG | Jackson ImmunoResearch, Suffolk, UK  111-035-045 | 1:10000, 5% milk in TBS-T 0.1% |
| α-Caspase1 | AbClonal,  Düsseldorf, Germany  A18646 | 1:1000, 5% milk in TBS-T 0.1% | HRP conjugated goat anti-rabbit IgG | Jackson ImmunoResearch  111-035-045 | 1:10000, 5% milk in TBS-T 0.1% |
| α-CD68 | Novus Biologicals, Littleton, CO, USA  NB100-683 | 1:1000, 5% BSA in TBS-T 0.1% | HRP conjugated goat anti-mouse IgG | Jackson ImmunoResearch  115-035-003 | 1:10000, 5% BSA in TBS-T 0.1% |
| α-CX43 | Novus Biologicals  NBP2-68678 | 1:1000, 5% BSA in TBS-T 0.1% | HRP conjugated goat anti-rabbit IgG | Jackson ImmunoResearch  111-035-045 | 1:10000, 5% BSA in TBS-T 0.1% |
| α-GFAP | Abcam, Cambridge,  UK  ab7260 | 1:25000, 5% milk in TBS-T 0.1% | HRP conjugated goat anti-rabbit IgG | Jackson ImmunoResearch  111-035-045 | 1:10000, 5% milk in TBS-T 0.1% |
| α-Iba1 | Novus Biologicals  NBP2-19019 | 1: 1000, 5% milk in TBS-T 0.1% | HRP conjugated goat anti-rabbit IgG | Jackson ImmunoResearch  111-035-045 | 1:10000, 5% milk in TBS-T 0.1% |
| α-IL-18 | Abcam  ab191860 | 1: 1000, 5% milk in TBS-T 0.1% | HRP conjugated goat anti-rabbit IgG | Jackson ImmunoResearch111-035-045 | 1:10000, 5% milk in TBS-T 0.1% |
| α-MAP2 | Novus Biologicals  NB600-1372 | 1:250  5 % BSA in TBS-T 0.1 % | HRP conjugated goat anti-mouse IgG | Jackson ImmunoResearch  115-035-003 | 1:10000  5 % BSA in TBS-T 0.1 % |
| α-NLRP3 | Cell Signaling,  Massachusetts, USA  15101 | 1:1000  5 % milk in TBS-T 0.1 % | HRP conjugated goat anti-rabbit IgG | Jackson ImmunoResearch  111-035-045 | 1:10000  5 % milk in TBS-T 0.1 % |
| α-p^[Ser536]^p65 | Santa Cruz Biotechnology, Dallas, Texas, USA  sc-136548 | 1: 1000, 5% BSA in TBS-T 0.1% | HRP conjugated goat anti-mouse IgG | Jackson ImmunoResearch  115-035-003 | 1:10000  5 % BSA in TBS-T 0.1 % |
| α-p65 | Santa Cruz Biotechnology  sc-8008 | 1: 1000, 5% BSA in TBS-T 0.1% | HRP conjugated goat anti-mouse IgG | Jackson ImmunoResearch  115-035-003 | 1:10000  5 % BSA in TBS-T 0.1 % |
| α-p50 | Abcam  ab32360 | 1: 1000, 5% milk in TBS-T 0.1% | HRP conjugated goat anti-rabbit IgG | Jackson ImmunoResearch  111-035-045 | 1:10000, 5% milk in TBS-T 0.1% |
| α-PSD95 | Santa Cruz Biotechnology  sc-32290 | 1: 1000, 5% milk in TBS-T 0.1% | HRP conjugated goat anti-mouse IgG | Jackson ImmunoResearch  115-035-003 | 1:10000  5 % milk in TBS-T 0.1 % |
| α-Synaptophysin | AbClonal  A6344 | 1: 1000, 5% milk in TBS-T 0.1% | HRP conjugated goat anti-rabbit IgG | Jackson ImmunoResearch  111-035-045 | 1:10000, 5% milk in TBS-T 0.1% |
| α-S100B | Genetex, Irvine, CA, USA  GTX129573 | 1: 1000  5 % BSA in TBS-T 0.1 % | HRP conjugated goat anti-rabbit IgG | Jackson ImmunoResearch  111-035-045 | 1:10000  5 % BSA in TBS-T 0.1 % |
| α-TLR4 | Invitrogen, Waltham, MA, USA  PA-23124 | 1: 1000  5 % milk in TBS-T 0.1 % | HRP conjugated goat anti-rabbit IgG | Jackson ImmunoResearch  111-035-045 | 1:10000  5 % milk in TBS-T 0.1 % |

BDNF: brain-derived neurotrophic factor; CD: cluster of differentiation; CX: connexin; BSA: bovine serum albumin; GFAP: glial fibrillary acidic protein; HRP: horseradish peroxidase; Iba1: ionized calcium-binding adapter molecule 1; IL: interleukin; MAP: microtubule associated protein; PSD: post synaptic density; TBS-T: tris buffered saline with tween 20; TLR: toll-like receptor.

**Table S2. List of primers and RT-PCR conditions**

| **Gene** | **Brand** | **Primer (5’ → 3’)** | | **Ann. (60°C)** | **Efficiency (%)** | **R^2^** |
| --- | --- | --- | --- | --- | --- | --- |
| CD11b | Bio-Rad, Hercules, CA,USA | Forward | N/A (Cod. qRnoCID0002800) | 60 | 94.0 | .998 |
|  |  | Reverse |  |  |  |  |
| FGF2 | Bio-Rad | Forward | N/A (Cod. qRnoCID0003540) | 60 | 96.0 | .999 |
|  |  | Reverse |  |  |  |  |
| GAPDH | Bio-Rad | Forward | N/A (Cod. qRnoCID0057018) | 60 | 96.0 | .999 |
|  |  | Reverse |  |  |  |  |
| GDNF | BioFab, Rome, Italy | Forward | CACCAGATAAACAAGCGGCG | 60 | 90.5 | .997 |
|  |  | Reverse | TCGTAGCCCAAACCCAAGTC |  |  |  |
| HPRT | BioFab | Forward | TCCCAGCGTCGTGATTAGTGA | 60 | 98.3 | .992 |
|  |  | Reverse | CCTTCATGACATCTCGAGCAAG |  |  |  |
| IL-6 | Sigma Aldrich | Forward | CAGAGTCATTCAGAGCAATAC | 60 | 100 | .998 |
|  |  | Reverse | CTTTCAAGATGAGTTGGATGG |  |  |  |
| IL-1β | Bio-Rad | Forward | N/A (Cod. qRnoCID0004680) | 60 | 98.0 | .999 |
|  |  | Reverse |  |  |  |  |
| TBP | BioFab | Forward | TGGGATTGTACCACAGCTCCA | 60 | 99.7 | .995 |
|  |  | Reverse | CTCATGATGACTGCAGCAAACC |  |  |  |
| TGF-β | Bio-Rad | Forward | N/A (qRnoCID0006448) | 60 | 102 | .999 |
|  |  | Reverse |  |  |  |  |
| TNF-α | Bio-Rad | Forward | N/A (qRnoCED0009117) | 60 | 98.0 | .999 |
|  |  | Reverse |  |  |  |  |

Ann.T: annealing temperature; CD: cluster of differentiation; FGF: fibroblast growth factor; GAPDH: glyceraldehyde-3-phosphate dehydrogenase; GDNF: glial derived neurotrophic factor; HPRT: hypoxanthine-guanine phosphoribosyltransferase; IL: interleukin; TBP: TATA-box binding protein; TGF: transforming growth factor; TNF: tumor necrosis factor.

**Table S3. List of antibodies and immunofluorescence conditions**

| **Primary antibody** | **Brand and Catalog #** | **Dilution** | **Secondary antibody** | **Brand and Catalog #** | **Dilution** |
| --- | --- | --- | --- | --- | --- |
| Rabbit α-GFAP | Abcam  ab7260 | 1: 1000, 5 % BSA in PBS-T 0.25% | FITC conjugated goat anti-rabbit IgG (H+L) | Jackson ImmunoResearch  111-095-003 | 1:400, 5% BSA in PBS-T 0.25% |
| Mouse α-GS | Millipore  MA, USA  MAB302 | 1:200, 5% BSA in PBS-T 0.25% | TRITC conjugated goat anti-mouse  IgG (H+L) | Jackson ImmunoResearch  115-025-003 | 1:400, 5% BSA in PBS-T 0.25% |
| Rabbit α-Iba1 | Wako, Osaka, Japan  019-19741 | 1:1000, 5 % BSA in PBS-T 0.25% | FITC conjugated goat anti-rabbit  IgG (H+L) | Jackson ImmunoResearch  111-095-003 | 1:200, 5% BSA in PBS-T 0.25% |
| Mouse α-NeuN | Abcam  ab104224 | 1:1000, 5 % milk in PBS-T 0.25% | TRITC conjugated goat anti-mouse  IgG (H+L) | Jackson ImmunoResearch  115-025-003 | 1:400, 5% BSA in PBS-T 0.25% |

BSA: bovine serum albumin; FITC: fluorescein isothiocyanate; GFAP: glial fibrillary acidic protein; GS: glutamine synthetase; Iba1: ionized calcium-binding adapter molecule 1; NeuN: neuronal nuclear protein; PBS-T: phosphate buffer saline with Triton; TRITC: tetramethyl rhodamine.

**Supplementary Results**

**Table S4. Results of repeated measures ANOVA for % variation of sucrose intake vs the baseline of CNT, FS-R and FS-V rats 24 and 48 h after acute FS**

| **Time (h)**  **after FS** | **CNT**  (Mean ± SEM) | **FS-R**  (Mean ± SEM) | **FS-V**  (Mean ± SEM) | **RM ANOVA result** |
| --- | --- | --- | --- | --- |
| 24 | 91.35 ± 5.18 | 90.77 ± 7.49 | 47.94 ± 2.94 | Stress: F (2, 17) = 86.61, ***p<*0,0001**  Time: F (1, 17) = 6.55, ***p=*0,020**  Interaction Stress*Time: F (2, 17)=4.09, ***p=*0,035** |
| 48 | 114.66 ± 3.28 | 98.39 ± 1.90 | 46.23 ± 4.51 |  |

**Table S5. Results for each target studied in the PFC of CNT, FS-R and FS-V rats 24 h after acute FS**

| **Target**  (alphabetical order) | **Molecular technique** | **CNT**  (Mean ± SEM) | **FS-R**  (Mean ± SEM) | **FS-V**  (Mean ± SEM) | **ANOVA result** |
| --- | --- | --- | --- | --- | --- |
| BDNF/total BDNF | WB | 1.00 ± 0.11 | 1.61 ± 0.06 | 0.65 ± 0.10 | F (2, 10) = 23.64, ***p=*0,0002** |
| Caspase1 | WB | 1.00 ± 0.15 | 0.79 ± 0.07 | 1.13 ± 0.17 | F (2, 11) = 1.10, *p*=0.37 |
| CD11b | PCR | 1.00 ± 0.15 | 0.96 ± 0.27 | 2.06 ± 0.12 | F (2, 12) = 6.07, ***p*=0.01** |
| CD68 | WB | 1.00 ± 0.11 | 1.50 ± 0.43 | 1.50 ± 0.22 | F (2, 8) = 1.59, *p*=0.26 |
| CX43 | WB | 1.00 ± 0.08 | 1.06 ± 0.29 | 1.56 ± 0.38 | F (2, 11) = 1.28, *p=*0.32 |
| FGF2 | PCR | 1.00 ± 0.14 | 0.95 ± 0.27 | 2.07 ± 0.12 | F (2, 12) = 6.16, ***p*=0.01** |
| GDNF | PCR | 1.00 ± 0.05 | 1.07 ± 0.05 | 0.87 ± 0.10 | F (2, 10) = 2.11, *p*=0.17 |
| GFAP | WB | 1.00 ± 0.03 | 1.25 ± 0.07 | 1.25 ± 0.07 | F (2, 12) = 6.34, ***p*=0.01** |
| GFAP^+^cells + GS^+^cells + GFAP^+^GS^+^cells | IF | 1636.33 ± 34.83 | 1624.05 ± 38.03 | 1697.23 ± 19.49 | F (2, 12) = 1.52, *p*=0.26 |
| GFAP^+^ cells n° of ends | IF | 11.53 ± 0.11 | 11.86 ± 0.41 | 11.72 ± 0.42 | F (2, 8) = 1.73, *p*=0.84 |
| GFAP^+^ cells length of branches | IF | 14.71 ± 0.74 | 15.33 ± 1.17 | 14.88 ± 0.68 | F (2, 8) = 0.11, *p*=0.89 |
| Iba1 | WB | 1.00 ± 0.06 | 0.75 ± 0.16 | 1.12 ± 0.24 | F (2, 11) = 1.17, *p*=0.34 |
| Iba1^+^cells | IF | 190.40 ± 17.84 | 202.79 ± 10.94 | 206.95 ± 9.83 | F (2, 12) = 0.42, *p*=0.67 |
| IL-6 | PCR | 1.00 ± 0.09 | 0.95 ± 0.11 | 1.08 ± 0.08 | F (2, 9) = 0.46, *p*=0.64 |
| IL-18 | WB | 1.00 ± 0.16 | 0.72 ± 0.27 | 2.04 ± 1.03 | F (2, 11) = 1.03, *p*=0.39 |
| IL-1β | PCR | 1.00 ± 0.27 | 0.94 ± 0.12 | 0.60 ± 0.05 | F (2, 14) = 2.49, *p*=0.12 |
| MAP2 | WB | 1.00 ± 0.06 | 1.90 ± 0.20 | 1.39 ± 0.20 | F (2, 11) = 7.34, ***p*=0.0094** |
| NeuN^+^cells | IF | 2712.56 ± 50.40 | 2789.41 ± 60.93 | 2605.71 ± 74.98 | F (2, 12) = 2.15, *p*=0.16 |
| NLRP3 | WB | 1.00 ± 0.08 | 1.02 ± 0.06 | 1.29 ± 0.10 | F (2, 11) = 0.50, *p*=0.62 |
| p50 | WB | 1.00 ± 0.02 | 1.17 ± 0.12 | 0.94 ± 0.18 | F (2, 9) = 0.95, *p*=0.42 |
| PSD95 | WB | 1.00 ± 0.10 | 0.92 ± 0.25 | 0.68 ± 0.06 | H (2) = 2.58, *p*=0.30 |
| p-CX43/  total CX43 | WB | 1.00 ± 0.10 | 0.98 ± 0.05 | 1.01 ± 0.07 | F (2, 11) = 0.02, *p*=0.98 |
| p^[Ser536]^p65 | WB | 1.00 ± 0.07 | 1.09 ± 0.09 | 0.91 ± 0.10 | F (2, 9) = 0.81, *p*=0.47 |
| p65 | WB | 1.00 ± 0.06 | 1.36 ± 0.14 | 1.12 ± 0.15 | F (2, 9) = 1.78, *p*=0.22 |
| Pro-Caspase1 | WB | 1.00 ± 0.13 | 0.92 ± 0.06 | 0.93 ± 0.07 | F (2, 11) = 0.17, *p*=0.84 |
| Synaptophysin | WB | 1.00 ± 0.02 | 0.82 ± 0.13 | 0.96 ± 0.16 | F (2, 9) = 0.60, *p*=0.57 |
| S100B | WB | 1.00 ± 0.14 | 0.88 ± 0.06 | 0.75 ± 0.14 | F (2, 11) = 1.12, *p*=0.36 |
| TGF-β | PCR | 1.00 ± 0.12 | 1.06 ± 0.07 | 0.85 ± 0.06 | F (2, 13) = 1.57, *p*=0.24 |
| TLR4 | WB | 1.00 ± 0.20 | 1.22 ± 0.14 | 0.83 ± 0.14 | F (2, 11) = 0.86, *p*=0.45 |
| TNF-α | PCR | 1.00 ± 0.34 | 2.03 ± 0.46 | 1.53 ± 0.45 | F (2, 11) = 1.03, *p*=0.39 |

**Table S6. Results for each target studied in the PFC of CNT, FS-R and FS-V rats 48 h after acute FS**

| **Target**  (alphabetical order) | **Molecular technique** | **CNT**  (Mean ± SEM) | **FS-R**  (Mean ± SEM) | **FS-V**  (Mean ± SEM) | **ANOVA result** |
| --- | --- | --- | --- | --- | --- |
| BDNF/total BDNF | WB | 1.00 ± 0.05 | 1.35 ± 0.10 | 1.04 ± 0.09 | F (2, 16) =3.79, ***p*=0.04** |
| Caspase1 | WB | 1.00 ± 0.05 | 1.03 ± 0.08 | 0.99 ± 0.06 | F (2, 9) =0.10, *p*=0.90 |
| CD11b | PCR | 1.00 ± 0.08 | 1.16 ± 0.07 | 1.41 ± 0.11 | F (2, 11) =5.46, ***p*=0.02** |
| CD68 | WB | 1.00 ± 0.10 | 0.95 ± 0.02 | 0.96 ± 0.08 | F (2, 15) =0.75, *p*=0.49 |
| CX43 | WB | 1.00 ± 0.04 | 1.02 ± 0.11 | 1.29 ± 0.13 | F (2, 16) =2.73, ***p*=0.02** |
| GDNF | PCR | 1.00 ± 0.18 | 1.23 ± 0.32 | 1.73 ± 0.45 | F (2, 12) = 1.08, *p*=0.37 |
| GFAP | WB | 1.00 ± 0.11 | 1.43 ± 0.28 | 1.42 ± 0.27 | F (2, 14) = 1.26, *p*=0.31 |
| GFAP^+^cells + GS^+^cells + GFAP^+^GS^+^cells | IF | 1628.98 ± 137.58 | 1953.16 ± 51.04 | 1693.46 ± 36.03 | F (2, 12) = 2.86, ***p*=0.0505** |
| GFAP^+^ cells n° of ends | IF | 9.94 ± 0.87 | 9.06 ± 0.19 | 9.20 ± 0.20 | F (2, 6) = 0.66, *p*=0.56 |
| GFAP^+^ cells length of branches | IF | 15.72 ± 1.02 | 16.40 ± 0.76 | 17.48 ± 0.72 | F (2, 6) = 0.81, *p*=0.49 |
| Iba1^+^cells | IF | 139.10 ± 6.99 | 225.92 ± 17.76 | 194.58 ± 3.99 | F (2, 12) = 15.24, ***p*=0.0005** |
| Iba1^+^ cells n° of ends | IF | 12.40 ± 1.71 | 11.13 ± 0.35 | 11.30 ± 0.53 | F (2, 9) = 0.42, *p*=0.67 |
| Iba1^+^ cells length of branches | IF | 19.53 ± 1.02 | 19.23 ± 0.64 | 19.88 ± 0.46 | F (2, 9) = 0.19, *p*=0.83 |
| Iba1^+^ cells diameter | IF | 4.61 ± 0.28 | 4.39 ± 0.40 | 4.63 ± 0.34 | F (2, 9) = 0.14, *p*=0.87 |
| Iba1 | WB | 1.00 ± 0.06 | 1.07 ± 0.14 | 0.95 ± 0.15 | F (2, 13) = 2.38, *p*=0.13 |
| IL-6 | PCR | 1.00 ± 0.17 | 0.72 ± 0.15 | 1.27 ± 0.40 | F (2, 8) = 1.06, *p*=0.39 |
| IL-18 | WB | 1.00 ± 0.12 | 1.09 ± 0.29 | 1.69 ± 0.07 | F (2, 12) = 5.32, ***p*=0.02** |
| IL-1β | PCR | 1.00 ± 0.06 | 0.93 ± 0.02 | 0.96 ± 0.06 | F (2, 12) = 0.49, *p*=0.62 |
| MAP2 | WB | 1.00 ± 0.09 | 1.14 ± 0.05 | 0.58 ± 0.06 | F (2, 16) = 13.07, ***p*=0.0004** |
| NeuN^+^cells | IF | 2618.86 ± 117.85 | 2703.49 ± 182.98 | 2565.14 ± 191.81 | F (2, 12) = 0.17, *p*=0.84 |
| NLRP3 | WB | 1.00 ± 0.08 | 1.02 ± 0.07 | 1.29 ± 0.08 | F (2, 12) = 0.77, *p*=0.49 |
| p^[Ser536]^p65 | WB | 1.00 ± 0.06 | 1.58 ± 0.21 | 1.50 ± 0.10 | F (2, 15) = 7.86, ***p*=0.0046** |
| p50 | WB | 1.00 ± 0.15 | 0.80 ± 0.18 | 0.98 ± 0.29 | F (2, 9) = 0.27, *p*=0.78 |
| p65 | WB | 1.00 ± 0.17 | 0.96 ± 0.18 | 1.59 ± 0.14 | F (2, 16) = 2.79, *p*=0.09 |
| Pro-Caspase1 | WB | 1.00 ± 0.05 | 0.96 ± 0.06 | 0.99 ± 0.06 | F (2, 9) = 0.11, *p*=0.89 |
| PSD95 | WB | 1.00 ± 0.08 | 1.01 ± 0.13 | 0.68 ± 0.08 | F (2, 13) = 4.78, ***p*=0.03** |
| S100B | WB | 1.00 ± 0.15 | 0.98 ± 0.14 | 1.56 ± 0.13 | F (2, 13) = 5.48, ***p*=0.02** |
| Synaptophysin | WB | 1.00 ± 0.11 | 1.00 ± 0.15 | 0.99 ± 0.14 | F (2, 17) = 0.84, *p*=0.45 |
| TGF-β | PCR | 1.00 ± 0.10 | 1.41 ± 0.08 | 1.25 ± 0.06 | F (2, 13) = 5.60, ***p*=0.02** |
| TNF-α | PCR | 1.00 ± 0.12 | 1.60 ± 0.09 | 2.17 ± 0.32 | F (2, 11) = 8.52, ***p*=0.006** |

**Table S7. Results for each target studied in the PFC of FS-V/Veh and FS-V/KET rats 48 h after acute FS**

| **Target**  **(**alphabetical order**)** | **Molecular technique** | **FS-V/Veh**  (Mean ± SEM) | **FS-V/KET** (Mean ± SEM) | **Student t-test result** |
| --- | --- | --- | --- | --- |
| BDNF | WB | 1.00 ± 0.04 | 1.11 ± 0.09 | t (12) = 1.08, *p*=0.30 |
| CD11b | PCR | 1.00 ± 0.11 | 0.76 ± 0.12 | t (7) = 4.21, ***p*=0.004** |
| CX43 | WB | 1.00 ± 0.06 | 0.88 ± 0.07 | t (10) = 2.61, ***p*=0.03** |
| GDNF | PCR | 1.00 ± 0.32 | 0.51 ± 0.04 | t (9) = 2.39, ***p*=0.04** |
| Iba1^+^cells | IF | 194.37 ± 2.67 | 139.55 ± 20.00 | t (5) = 3.22, ***p*=0.02** |
| IL-18 | WB | 1.00 ± 0.05 | 0.77 ± 0.08 | t (9) = 2.37, ***p*=0.04** |
| MAP2 | WB | 1.00 ± 0.09 | 1.51 ± 0.28 | t (10) = 2.58, ***p*=0.02** |
| p-CX43/total CX43 | WB | 1.00 ± 0.02 | 1.00 ± 0.10 | t (8) = 0.04, *p*=0.96 |
| p^[Ser536]^p65 | WB | 1.00 ± 0.06 | 0.79 ± 0.04 | t (10) = 2.70, ***p*=0.02** |
| p65 | WB | 1.00 ± 0.07 | 1.32 ± 0.18 | t (12) = 1.63, *p*=0.13 |
| PSD95 | WB | 1.00 ± 0.13 | 1.72 ± 0.75 | t (10) = 1.37, *p*=0.20 |
| S100B | WB | 1.00 ± 0.05 | 0.84 ± 0.05 | t (12) = 2.25, ***p*=0.04** |
| TGF-β | PCR | 1.00 ± 0.05 | 1.00 ± 0.05 | t (8) = 0.08, *p*=0.93 |
| TLR4 | WB | 1.00 ± 0.13 | 1.15 ± 0.23 | t (12) = 0.57, *p*=0.58 |
| TNF-α | PCR | 1.00 ± 0.10 | 0.61 ± 0.08 | t (5) = 3.30, ***p*=0.02** |

**Table S8. Results for each target studied in the PFC of FS-R/Veh and FS-R/KET rats 48 h after acute FS**

| **Target**  (alphabetical order) | **Molecular technique** | **FS-R/Veh** (Mean ± SEM) | **FS-R/KET** (Mean ± SEM) | **Student t-test result** |
| --- | --- | --- | --- | --- |
| BDNF | WB | 1.00 ± 0.05 | 0.92 ± 0.03 | t (8) = 1.32, *p*=0.22 |
| CD11b | PCR | 1.00 ± 0.09 | 0.58 ± 0.05 | t (9) = 4.01, ***p*=0.003** |
| CX43 | WB | 1.00 ± 0.10 | 0.87 ± 0.16 | t (6) = 0.64, *p*=0.55 |
| GDNF | PCR | 1.00 ± 0.17 | 0.48 ± 0.20 | t (8) = 1.34, *p*=0.21 |
| Iba1^+^cells | IF | 226.40 ±17.58 | 180.28 ± 7.54 | t (4) = 2.41, *p*=0.07 |
| IL-18 | WB | 1.00 ± 0.24 | 1.17 ± 0.58 | t (6) = 0.27, *p*=0.79 |
| MAP2 | WB | 1.00 ± 0.21 | 1.07 ± 0.18 | t (8) = 0.26, *p*=0.79 |
| p-CX43/total CX43 | WB | 1.00 ± 0.05 | 1.05 ± 0.05 | t (6) = 0.05, *p*=0.96 |
| p^[Ser536]^p65 | WB | 1.00 ± 0.07 | 1.03 ± 0.04 | t (5) = 0.20, *p*=0.85 |
| p65 | WB | 1.00 ± 0.01 | 0.95 ± 0.05 | t (4) = 1.12, *p*=0.33 |
| PSD95 | WB | 1.00 ± 0.12 | 1.06 ± 0.23 | t (5) = 0.25, *p*=0.81 |
| S100B | WB | 1.00 ± 0.09 | 0.99 ± 0.04 | t (8) = 0.09, *p*=0.93 |
| TGF-β | PCR | 1.00 ± 0.05 | 0.86 ± 0.03 | t (8) = 2.07, *p*=0.07 |
| TLR4 | WB | 1.00 ± 0.07 | 1.04 ± 0.12 | t (6) = 0.25, *p*=0.81 |
| TNF-α | PCR | 1.00 ± 0.05 | 0.90 ± 0.07 | t (6) = 1.23, *p*=0.26 |

**Supplementary Figure 1**


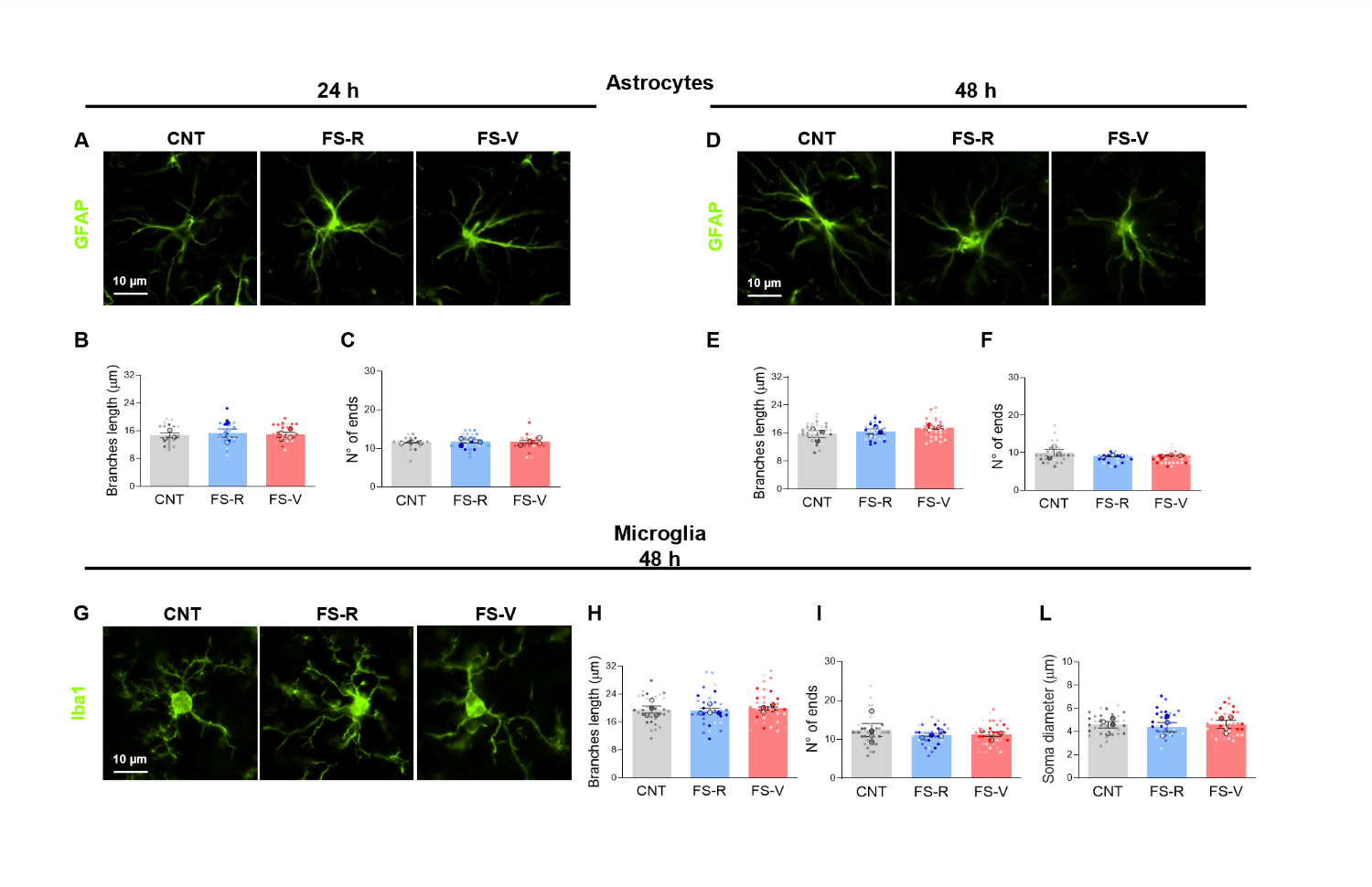


**Legend to Supplementary Figure 1**

Representative photomicrographs of PFC sections from CNT, FS-R and FS-V rats, stained for GFAP and Iba1.The length of the processes and the number of endpoints were measured in GFAP-immunopositive astrocytes 24 h (**A-C**) and 48 h (**D-F**) after FS. The length of processes, the number of endpoints and the soma diameter were measured in microglial cells 48 h after FS (**G-L**). Graphs show means ± sem of N=3-4 rats/group, n=3-4 slices/rat, n=2-3 images/slice, taken under a 20X objective. One-way ANOVA. The values of each individual cell (small dots) were used to obtain the group mean (large dots) and sem.

**Reference**

1. Bonifacino T, Mingardi J, Facchinetti R, Sala N, Frumento G, Ndoj E et al. Changes at glutamate tripartite synapses in the prefrontal cortex of a new animal model of resilience/vulnerability to acute stress. Transl Psychiatry 2023; 13(1)**:** 62.

2. Pfaffl MW. A new mathematical model for relative quantification in real-time RT-PCR. Nucleic Acids Res 2001; 29(9)**:** e45.

3. Torres-Platas SG, Comeau S, Rachalski A, Bo GD, Cruceanu C, Turecki G et al. Morphometric characterization of microglial phenotypes in human cerebral cortex. J Neuroinflammation 2014; 11**:** 12.
